# Frontal Release Signs and Future Decline in Research Participants With Intact Cognition

**DOI:** 10.1001/jamanetworkopen.2026.17060

**Published:** 2026-06-05

**Authors:** Lauren G. Bojarski, Gregory A. Jicha, Elif Pinar Coskun, Frederick A. Schmitt, Linda Van Eldik, Erin L. Abner

**Affiliations:** 1 University of Kentucky Sanders-Brown Center on Aging, Lexington; 2 Department of Neurology, University of Kentucky Medical Center, Lexington; 3 Epidemiology and Environmental Health, University of Kentucky, Lexington; 4 Now with Department of Neurology, West Virginia University Rockefeller Neuroscience Institute, Morgantown; 5 Neuroscience, University of Kentucky, Lexington

## Abstract

**Importance:**

Frontal release signs (FRS) are primitive reflexes that regress with brain maturation and reappear in the setting of brain injury or neurodegeneration. Despite their association with dementia, the prognostic utility of this finding in the setting of early cognitive decline and preclinical disease is poorly understood.

**Objective:**

To evaluate the association of FRS with future cognitive decline in research participants.

**Design, Setting, and Participants:**

This cohort study examined the presence of FRS (from 2005-2024) among research cohort participants with intact cognition or mild impairment at study baseline. The study used single-center data collection of longitudinal research cohort data through the Uniform Data Set at the University of Kentucky Alzheimer Disease Research Center (UK ADRC). Participants aged 70 years or older at first assessment were identified at baseline enrollment as having intact cognition or mild impairment based on consensus report and had at least 2 total annual assessments that included standard protocols involving assessment of FRS on neurological exam from the UK ADRC.

**Exposure:**

Participants were noted to be FRS positive or FRS negative.

**Main Outcomes and Measures:**

The main outcome of interest was incident dementia based on annual consensus diagnosis. Cause-specific hazard models were used to estimate the risk of transition to dementia when at least 2 FRS were present (FRS positive) at baseline vs when 0 or 1 FRS were present (FRS negative), and linear mixed models were used to investigate the association of baseline FRS positivity with cognitive performance over time.

**Results:**

A total of 873 participants (mean [SD] age, 76.9 [5.6] years; 527 female [60.4%]) were included, with a mean (SD) of 16.1 (2.8) years of education. The prevalence of FRS positivity at baseline was 59 of 672 participants (8.8%) with intact cognition and 48 of 201 participants (23.9%) with mild impairment. FRS positivity was associated with an increased risk of dementia for participants who were cognitively intact; in this group, 15 of 59 participants who were FRS positive (25.4%) progressed to dementia compared with 89 of 613 participants (14.5%) who were FRS negative (hazard ratio, 1.78; 95% CI, 1.02-3.09).

**Conclusions and Relevance:**

This study’s findings suggest that FRS may be a quick, noninvasive, and low-cost adjunct to neurological examination with prognostic utility in the earliest stages of cognitive decline.

## Key Points

**Question:**

Are frontal release signs (FRS), such as snout, grasp, and palmomental reflexes, associated with future cognitive decline?

**Findings:**

In this cohort study that included 873 older participants with intact cognition or mild impairment, having 2 or more FRS (FRS positive) was associated with increased hazard of dementia for those who were cognitively intact at baseline, with more participants who were FRS positive progressing to dementia during follow-up compared with participants who were not FRS positive.

**Meaning:**

These findings suggest that FRS should serve as an adjunct to other cognitive screening measures in the clinic.

## Teaser

This cohort study investigates the association of frontal release signs with future cognitive decline in older individuals with intact cognition.

## Introduction

Frontal release signs (FRS), also known as primitive or regressive reflexes, are clinical neurologic signs that are present at birth and gradually disappear as the brain matures in early life.^1,2,3,4,5^ The reemergence of these reflexes in later life is associated with brain injury or degeneration, as can be seen in late-life dementia.^1,2,3,4,5,6,7,8,9,10,11,12^ FRS include the glabellar tap (Myerson sign), grasp, rooting, snout, and palmomental reflexes.^4^ They are easy to test (ie, quick, noninvasive, and low cost) and are classically considered part of the complete neurologic examination.^4^ However, despite the historical significance of FRS, the sensitivity and specificity of individual FRS in identifying brain injury or degeneration are quite low^1,2,3,4,5,6,7,8,9,10,11,12^; in a meta-analysis^13^ of 29 studies, persons with dementia had up to a 16-fold higher probability of FRS, with the grasp reflex associated the highest risk for dementia. However, a single FRS may be observed in individuals who are neurologically intact at rates as high as 20%.^1,2,3,4,5,8,9,11,12,14^ In contrast, 2 or more FRS are rarely reported in individuals without brain injury or degenerative dementia.^2,6,9,11,12^

Previous research has demonstrated that applying a threshold of multiple FRS in an individual greatly increases the specificity of FRS for neurologic dysfunction, although the sensitivity remains quite low.^2,6,9,11,12,15^ This has been demonstrated in Alzheimer disease (AD) and vascular dementia, and so while the specificity of 2 or more FRS for brain injury or degeneration has been reported as high as 93%, diagnostic specificity for disease etiology has limited their utility as a diagnostic tool in standard clinical practice.^2,6,9,11,12^

The advent of biomarkers for neurodegenerative diseases that augment traditional neuropsychological testing has decreased use of the clinical neurologic examination in the diagnosis of late-life dementia, such as AD, that may not be associated with focal neurologic deficits or clinical examination findings.^16,17,18,19,20,21,22,23,24,25,26,27,28^ Biomarkers, including spinal fluid assays, genetic testing, advanced imaging modalities like volumetric magnetic resonance imaging and molecular positron emission tomography imaging, and most recently blood-based assays,^29^ are able to identify disease etiologies that can be associated with cognitive decline. However, these are frequently invasive and always costly, thus limiting their widespread use as screening measures for the spectrum of cognitive impairment.^30,31^ As the prevalence of dementia continues to increase worldwide, the importance of early detection is increasing given the newest developments in management and treatment to maintain quality of life.^32^ While many researchers and clinicians are continuously working to develop more-accurate, low-cost, and noninvasive screening tools for early cognitive decline, such as blood-based biomarkers for AD,^30,31,33,34^ the utility of the standard neurologic examination with a quick and easy assessment of FRS remains to be fully explored for this purpose and is evaluated in this study.

## Methods

### Participants

We queried the database of the University of Kentucky Alzheimer Disease Research Center (UK-ADRC) longitudinal research cohort to characterize FRS across a large population of prospectively evaluated research volunteers spanning the cognitive continuum from intact cognition to mild cognitive impairment (MCI) and dementia. Cohort exclusion criteria at entry included signs of neurologic disease, such as vascular insults and parkinsonism. Participants enrolled in the UK-ADRC longitudinal cohort are initially evaluated and then followed up approximately annually until death.^35^ Since September 1, 2005, all participants have been evaluated with a standard protocol used at all federally funded ADRCs, the National Alzheimer Coordinating Center Uniform Data Set (UDS).^36^ Participant demographic information was collected at the baseline UDS interview and included participant-reported sex (male or female) and race (American Indian or Alaska Native, Asian, Black or African American, White, or other) and ethnicity (Hispanic or non-Hispanic). Race and ethnicity were assessed to identify further disparities in health. Each annual UDS evaluation includes a medical history, a physical examination, and an extensive battery of neuropsychological tests of cognitive status. Additionally, the UK-ADRC performs a detailed neurological evaluation, including FRS, at all annual assessments; the details of this evaluation have been described previously.^37^ The Strengthening the Reporting of Observational Studies in Epidemiology (STROBE) reporting guideline for cohort studies was used for reporting this study.

Participants included in the study were age 70 years or older at their first UDS assessment, had at least 2 UDS assessments, had a clinical diagnosis of intact cognition (672 participants) or no more than mild impairment (201 participants) at the first UDS assessment, and were evaluated between September 1, 2005, and November 30, 2024 (873 participants total). All research activities were approved by the University of Kentucky Institutional Review Board, and all participants provided written informed consent or if unable to provide consent then by their proxy.

### Cognitive Diagnoses

Syndromic cognitive diagnoses of intact (or normal) cognition, MCI, impaired but not MCI, and dementia were assigned to participants at each annual visit using standard clinical criteria and consensus review according to UDS procedures. No participants with dementia at baseline were included. Baseline diagnoses were categorized as “intact cognition” (diagnosis of normal) or “mild impairment” (diagnosis of MCI or impaired not MCI).

### Neuropsychological Testing

The UDS includes a standardized neuropsychological testing battery. Our study period spans 2 version of the battery: UDS 2.0 (2005-2015) and UDS 3.0 (2015-2024).^38^ Tests common to both batteries include Animal Naming, Vegetable Naming, and Trail Making Tests A (TMTA) and B (TMTB). For UDS 3.0, the Mini-Mental State Examination (MMSE) was replaced by the Montreal Cognitive Assessment, the Wechsler Logical Memory test was replaced by the Craft Story 21 test, the Boston Naming Test was replaced by the Multilingual Naming Test, and Digit Span Forward and Backward were replaced by Number Span Forward and Backward. For analysis, scores from UDS 3.0 tests were recoded to equivalent UDS 2.0 scores based on the crosswalk published by the National Alzheimer’s Coordinating Center (NACC).^38^ Then, we converted individual test scores into *z* scores normed to NACC participants who were cognitively intact per Weintraub et al,^39^ then converted *z* scores and to mean domain scores (memory, attention, executive, and language) according to Hayden et al.^40^ The executive domain *z* score, which comprised TMTA and TMTB, was multiplied by −1 so that lower *z* scores indicated worse performance.

### Frontal Release Signs

At each annual assessment, study neurologists examined participants for the presence of the following FRS: grasp (bilateral), palmomental (bilateral), Gegenhalten (bilateral), snout, glabellar, suck, and jaw jerk. The number of positive reflexes was summed, and participants with at least 2 FRS were considered FRS positive; participants with 1 or 0 FRS were considered FRS negative. For this study, participants were considered FRS positive at all assessments after the first appearance of FRS positivity.

### General Motor Function

All participants were assessed with the Unified Parkinson Disease Rating Scale (UPDRS).^41^ To assess the possibility of confounding of the association of FRS and dementia risk by impaired motor function, we examined 2 parameters from the UPDRS: whether any items on the UPDRS were rated abnormal (yes or no) and gait function (possible responses: normal, walks slowly, walks with difficulty, severe disturbance of gait, cannot walk at all, and untestable).

### *APOE* Genotype

Participant blood samples were shipped from the UK-ADRC to the National Centralized Repository for Alzheimer Disease. *APOE* genotyping was performed here.

### Statistical Analysis

Descriptive statistics were used to assess differences in baseline demographic, clinical, and genetic variables between participants who were FRS negative and FRS positive at baseline (ie, first UDS) within the 2 cognitive groups. To evaluate the association between FRS and dementia risk, we fit cause-specific hazard models to the data to account for the competing risk of death before dementia. Results of cause-specific hazard models are interpreted as the hazard of the event of interest among participants who have not yet experienced any event (here, dementia diagnosis or death before dementia).^42^ Models used study baseline as time zero and adjusted for baseline cognitive diagnosis, MMSE score, age, sex, years of education, and abnormal gait. For analysis, gait was dichotomized as normal or abnormal. Finally, a cross-product interaction between baseline diagnosis and FRS status was included to account for differences in baseline risk of dementia. The proportional hazards assumption was assessed with the Grambsch and Therneau test. Participants who did not develop dementia were censored at their last study visit. Death before dementia was assumed to be informative censoring (risk of future dementia is known to be zero), while all other censoring mechanisms were assumed to be uninformative (risk of future dementia is assumed to be the same as among participants who remained under follow-up).

Linear mixed models with visit age as the time scale (adjusted for age, sex, and education) were used for analyses of longitudinal cognitive domain scores. Estimation was performed using restricted maximum likelihood, and robust standard errors were used. The within-participant correlation structure was assumed to be first-order autoregressive. Summary scores based on UDS 2.0 cognitive tests were used as independent outcomes.^39^ The interaction term for time (measured as visit age) and FRS positivity was used to assess group differences in change in the mean scores over time. These analyses were conducted separately within groups defined by participant baseline cognitive status. All statistical analyses were performed using SAS statistical software version 9.4 TS1M7 (SAS Institute). All statistical tests were 2-sided. Statistical significance was set at *P* < .05.

For both sets of analyses (survival and longitudinal), models were fit with and without *APOE4* status included due to missingness in *APOE* genotype (63 of 873 participants [7.2%]). This missingness was assumed to be noninformative because reasons for missingness were administrative and not related to participant decisions. In other words, participants did not decline *APOE* testing due to concerns about what the testing would reveal.

## Results

This cohort included 873 participants (mean [SD] age, 76.9 [5.6] years; 527 female [60.4%]; 7 Asian American [0.8%], 83 Black [9.5%], and 783 White [89.7%]), with a mean (SD) of 16.1 (2.8) years of education, among whom 251 participants (28.8%) were carriers of *APOE4*. Demographic and clinical variables by cognitive diagnosis and the presence or absence of 2 or more FRS (FRS positivity) at baseline are presented in Table 1. The prevalence of FRS positivity at baseline was 59 of 672 participants (8.8%) with intact cognition (mean [SD] number of FRS, 2.41 [0.67] FRS), and 48 of 201 participants (23.9%) with mild impairment (mean [SD] number of FRS, 2.79 [1.22] FRS) (Table 1). The most common FRS were palmomental and glabellar FRS (Figure). Within groups defined by baseline intact cognition or mild impairment, participants who were FRS positive and FRS negative were statistically similar on study follow-up time, age, sex, education, *APOE4* carriership, gait performance, and overall presence or absence of any parkinsonian features. Among participants with intact cognition, unadjusted mean performance in attention, executive, and language function was significantly poorer among the FRS-positive group. Among participants with mild impairment, there was no difference in domains (Table 1). Means of individual cognitive tests are presented in the eTable in Supplement 1.

**Table 1.** Study Population Demographic and Clinical Data by Diagnostic Grouping and FRS Positivity^a^

Characteristic	Participants, No. (%) (N = 873)			
	Intact cognition		Mild impairment	
	FRS negative (n = 613)	FRS positive (n = 59)	FRS negative (n = 153)	FRS positive (n = 48)
Follow-up, mean (SD), y	7.5 (4.7)	7.2 (4.1)	4.0 (2.8)	4.4 (4.0)
Baseline age, mean (SD), y	76.2 (5.2)	77.9 (6.8)	78.6 (5.9)	79.3 (5.5)
Education, mean (SD), y	16.1 (2.8)	16.8 (2.6)	15.8 (3.0)	15.9 (3.1)
Sex				
Male	223 (36.4)	22 (37.3)	77 (50.3)	24 (50.0)
Female	390 (63.6)	37 (62.7)	76 (49.7)	24 (50.0)
Race and ethnicity^b^				
Asian American	3 (0.5)	0	3 (2.0)	1 (2.1)
Black or African American	54 (8.8)	7 (11.9)	18 (11.8)	4 (8.3)
White	556 (90.7)	52 (88.1)	132 (86.3)	43 (89.6)
*APOE* ε4				
≥1 ε4	157 (25.6)	18 (30.5)	56 (36.6)	20 (41.7)
Missing	41 (6.7)	2 (3.4)	17 (11.1)	3 (6.3)
No. of FRS, mean (SD)	0.086 (0.28)	2.41 (0.67)	0.17 (0.38)	2.79 (1.22)
Any parkinsonian signs	176 (28.7)	23 (39.0)	43 (28.1)	20 (41.7)
Gait performance				
Normal	573 (93.5)	53 (89.8)	136 (88.9)	38 (79.2)
Walks slowly or with difficulty	35 (5.7)	5 (8.5)	15 (9.8)	10 (20.8)
Severe disturbance	2 (0.3)	1 (1.7)	1 (0.7)	0
Cannot walk at all	1 (0.2)	0	1 (0.7)	0
Untestable	2 (0.3)	0	0	0
Cognitive domain *z* score, mean (SD)				
Memory	−0.14 (0.92)	−0.11 (0.98)	−1.40 (0.99)	−1.10 (1.00)
Attention	0.022 (0.77)	0.011 (0.87)	−0.32 (0.80)	−0.45 (0.73)
Executive	−0.18 (0.82)	−0.46 (0.98)	−1.01 (1.17)	−1.30 (1.30)
Language	0.053 (0.68)	−0.13 (0.60)	−0.69 (0.71)	−0.73 (0.80)

Abbreviation: FRS, frontal release signs.

^a^ FRS positivity was defined as the presence of 2 or more FRS at baseline. Participants with 1 or 0 FRS were defined as FRS negative. Characteristics were compared within diagnostic groups (intact cognition and mildly impaired) with Student *t* test or χ^2^ statistics.

^b^ The ethnicity for all participants was non-Hispanic.

**Figure.**
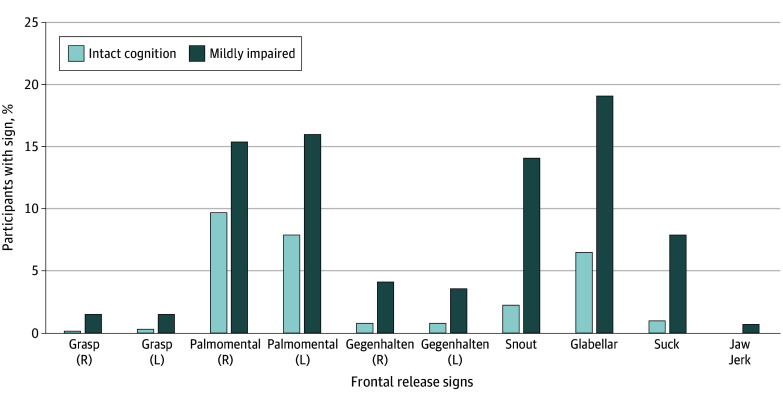
Bar Graph of Participants With Frontal Release Signs by Cognitive Status Percentages are given of included University of Kentucky Alzheimer Disease Research Center participants showing individual frontal release signs at baseline by cognitive status. L indicates left; R, right.

FRS positivity was associated with an increased hazard of dementia for individuals who were initially cognitively intact (Table 2). Among these participants, 15 of 59 participants who were FRS positive (25.4%) progressed to dementia during follow-up compared with 89 of 613 participants (14.5%) who were FRS negative (hazard ratio, 1.78; 95% CI, 1.02-3.09). Additionally, 15 participants who were FRS positive (25.4%) and 174 participants who were FRS negative (28.4%) died without a dementia diagnosis. For participants who were initially cognitively intact, the adjusted cause-specific hazard ratio (HR) for dementia for FRS-positive vs FRS-negative groups was 1.80 (95% CI, 1.04-3.13), while the adjusted cause-specific HR for death without dementia was 0.95 (95% CI, 0.56-1.63).

**Table 2.** Cause-Specific Hazard Model Results

Outcome	Baseline cognition	Participants, No. (N = 873)	Events, No.	HR (95% CI)^a^
Dementia				
*APOE* e4 included	Intact	672	104	1.80 (1.04-3.13)
*APOE* e4 not included	Impaired	201	104	1.13 (0.74-1.74)
Death				
*APOE* e4 included	Intact	672	189	0.95 (0.56-1.63)
*APOE* e4 not included	Impaired	201	41	0.44 (0.20. 0.97)

Abbreviations: HR, hazard ratio.

^a^ All models adjusted for baseline age, sex, years of education, baseline Mini-Mental State Examination, and an indicator for abnormal gait.

FRS positivity was not associated with the hazard of dementia for participants who were initially mildly impaired (Table 2). Among these participants, 30 of 48 participants (62.5%) who were FRS positive progressed to dementia during follow-up compared with 74 of 153 participants (48.4%) who were FRS negative; 8 participants who were FRS positive (16.7%) died without a dementia diagnosis compared with 33 participants who were FRS negative (21.6%). The adjusted HR for dementia for FRS-positive vs FRS-negative groups was 1.13 (95% CI, 0.74-1.74), while for the outcome of death without dementia, the adjusted HR was 0.44 (95% CI, 0.20-0.97).

Linear mixed model analyses adjusted for baseline age, sex, and education yielded limited evidence of different rates of change in cognitive performance by FRS positivity but again among participants who were initially cognitively intact (Table 3). Specifically, mean memory and executive *z* scores worsened slightly but significantly faster over time; for example, the mean change in memory z score with *APOE4* status included was −0.025 (95% CI, −0.040 to −0.011) among participants who were initially cognitively intact. For participants with baseline impairment, there were no significant differences. For baseline intact cognition and baseline impairment, sensitivity analyses revealed no substantive changes in results due to inclusion or exclusion of participants with missing *APOE* (Table 2 and Table 3).

**Table 3.** Linear Mixed Model Analyses for Cognitive Performance^a^

Cognitive status	Cognitive domain β coefficient (95% CI)			
	Memory	Attention	Executive	Language
Intact cognition				
*APOE* e4 included	−0.025 (−0.039 to −0.011)	0.001 (−0.009 to 0.012)	−0.017 (−0.030 to −0.004)	−0.0009 (−0.009 to 0.007)
*APOE* e4 not included	−0.025 (−0.040 to −0.011)	0.002 (−0.009 to 0.012)	−0.017 (−0.030 to −0.004)	−0.001 (−0.009 to 0.007)
Mild impairment				
*APOE* e4 included	0.014 (−0.012 to 0.041)	−0.007 (−0.027 to 0.013)	0.024 (−0.013 to 0.061)	0.018 (−0.002 to 0.038)
*APOE* e4 not included	0.010 (−0.017 to 0.037)	−0.004 (−0.024 to 0.016)	0.031 (−0.007 to 0.070)	0.015 (−0.005 to 0.035)

^a^ All models adjusted for baseline age, sex, and years of education. Results presented are β coefficients (95% CI) for the visit age × frontal release signs interaction and may be interpreted as the mean change in the domain *z* score per 1-year increase in age for participants with vs without frontal release signs positivity at baseline.

## Discussion

In this retrospective cohort study of participants evaluated for FRS and cognitive performance, having 2 or more FRS was associated increased risk of future cognitive decline among older adults with intact cognition, with nearly twice the risk of dementia in this group.

These data further demonstrated that the presence of 2 or more FRS was associated with lower baseline cognitive performance on some measures for participants with intact cognition and in some cases was associated with a faster rate of decline. Notably, for participants with baseline intact cognition, cognitive tests showing association with FRS-positive status included MMSE, Logical Memory, and Trail Making Test B. These tests are highly dependent on working memory, attention, speed of processing, and motor control, which are subserved by medial frontal cortical and subcortical networks. Tests dependent on language and executive function dependent on lateral frontal and temporal cortical networks showed no such association with FRS-positive status. These data may provide insight into the neuroanatomical circuitry involved in the suppression and reemergence of FRS as one progresses along the cognitive continuum, suggesting a medial frontal, subcortical involvement rather than a diffuse cortical process.

In our clinical experience, all individuals living long enough to progress to the end stages of dementia (bedbound with full dependence for all basic activities of daily living) develop the full repertoire of FRS.^6,43^ While supporting an argument for intrinsic resistance in the involved neuronal circuitry, this observation does not preclude the possibility that as the degenerative process spreads, the damage extrinsic to medial frontal and subcortical circuitry implicated previously plays a role in the reemergence of FRS in many individuals living to the final stages of dementia.

Estimation of cognitive decline in individuals with intact cognition and in those with MCI has been a central focus in the development of diagnostic biomarkers for AD and other degenerative dementias.^16,17,18,19,20,21,22,23,24,25,26,27,28^ Data from composite neuropsychological measures have demonstrated a 30% improvement in estimation of MCI converting to dementia over a 3-year period.^44,45^ Imaging data can improve the estimation of individuals transitioning from MCI to dementia by 76% over a 4-year period.^45,46,47,48,49,50,51^ Given our study’s findings, the utility of the clinical neurologic examination in the estimation of imminent cognitive decline for individuals with intact cognition should not be dismissed.

To our knowledge, there exists only 1 other study in the literature that has evaluated the longitudinal prognostic implications of FRS in participants who are cognitively intact. The Maastricht Aging Study (MAS) did not find any association of FRS with cognitive outcomes in 470 adults older than age 50 years who were cognitively intact and followed up prospectively at 3 and 6 years.^14^ This study differed from ours in several key respects. The neuropsychological test battery with the MAS assessed executive and language function predominantly, 2 cognitive domains we found not to be associated with FRS. The MAS study found an association between age and the presence of FRS, yet less than one-third of participants were older than age 66 years (136 participants), compared with the much larger numbers of older individuals who were cognitively intact in our study (672 participants). The 3-year interval between baseline and subsequent evaluations in MAS may have allowed transitions to escape capture or the emergence of incident FRS to be seen and incorporated into the analysis. In addition, more than 30% of the sample was lost to follow up, and while discussion of analyses looking at the MCI homologue labeled “cognitive impairment no dementia” were included, it is not ascertainable whether any of their sample transitioned to a more advanced clinical state that would allow such interpretation. Our study may overcome these limitations using a robust cohort of individuals who were at risk with more advanced age, examined with a more comprehensive neuropsychological test battery and FRS examination at regular, more frequent intervals. This allowed capture of transitions to MCI or dementia that are the primary outcomes of our study.

The examination of FRS in an individual patient can be accomplished in less than 2 minutes within the context of a standard clinical examination. With the recent US Food and Drug Administration approval for blood-based biomarkers for Alzheimer disease,^29^ FRS positivity may serve to be an excellent adjunct tool in screening individuals with intact cognition once treatment is developed and approved for those with preclinical AD. While outside the parameters of this study, future avenues may include evaluation of blood-based biomarkers associated with Alzheimer disease and their correlation with FRS to delineate further associations.

### Limitations

This study has limitations, including the use of a single ADRC data. While our site has standardized procedures and collection of data on FRS, these can be elicited in many subtly different but potentially important ways and may be absent secondary to human error. It is unclear if all methods of eliciting FRS would yield comparable results. Additionally, we note that our population largely comprises individuals with advanced age and higher education, with relatively homogenous racial demographics. Additionally, given that this study spanned 2 versions of the UDS battery, there is not a perfect equivalence between scores (eg, Logical Memory vs Craft Story 21 are imperfectly correlated) and this could have affected our longitudinal analyses.

Of note, FRS lack sensitivity, and relying on FRS alone would be suboptimal for a cognitive screening measure; many people who are at risk with intact cognition, as well as those with MCI or overt dementia, may be missed. Furthermore, the presence or absence of FRS provides no useful prognostic information once a diagnosis of dementia is made. The examination of FRS should therefore be used preferentially in individuals with intact cognition. It should be considered a useful component of a more comprehensive examination or screen for cognitive decline, rather than a stand-alone evaluative tool for the practicing clinician.

## Conclusions

Ultimately, this cohort study’s findings suggest that the widespread examination of FRS in the aging population has ecological validity as a tool to augment preclinical detection of impending cognitive decline.^4^ While not supplanting neuropsychological testing or the use of diagnostic biomarkers, FRS may serve as accessory evidence heightening clinical suspicion of imminent cognitive decline as we search for more effective and practical methods to screen the aging population at risk for development of a degenerative dementia.

## Notes

Supplement 1. **eTable.** Raw Cognitive Test Scores

Supplement 2.
Data Sharing Statement
