# Supplementary material for: Frontal Release Signs and Future Decline in Research Participants With Intact Cognition
Source: JAMA Netw Open. 2026 Jun 5;9(6):e2617060. doi: 10.1001/jamanetworkopen.2026.17060 (PMC13241946; doi:10.1001/jamanetworkopen.2026.17060)
Supplement: Supplement 1. — eTable. Raw Cognitive Test Scores [file jamanetwopen-e2617060-s001.pdf]

Supplemental Online Content

Bojarski LG, Jicha GA, Coskun EP, Schmitt FA, Van Eldik L, Abner EL. Frontal release signs and future decline in research participants with intact cognition. *JAMA Netw Open*. 2026;9(6):e2617060. doi:10.1001/jamanetworkopen.2026.17060

eTable. Raw Cognitive Test Scores

This supplemental material has been provided by the authors to give readers additional information about their work.

© 2026 Bojarski LG et al. *JAMA Network Open*.

eTable. Raw Cognitive Test Scores

	Intact Cognition		Mild Impairment	
	FRS- (n=613)	FRS+ (n=59)	FRS- (n=153)	FRS+ (n=48)
**Cognitive test scores (mean [sd])**				
**MMSE**	29.1 (1.1)	29.0 (1.2)	27.1 (2.2)	27.3 (2.2)
**Logical Memory I**	13.2 (3.7)	13.5 (4.1)	8.4 (4.0)	9.5 (4.0)
**Logical Memory II**	12.1 (4.1)	12.0 (4.2)	6.6 (4.4)	8.0 (4.0) [b]
**Digits Forward Trials**	9.4 (1.8)	8.7 (2.1) [b]	8.4 (2.0)	8.2 (1.9)
**Digits Backward Trials**	7.0 (2.0)	6.8 (2.1)	5.7 (2.0)	5.4 (1.8)
**Animal Naming**	20.0 (5.3)	19.8 (5.7)	16.1 (4.4)	15.4 (5.2)
**Vegetable Naming**	14.5 (4.0)	12.5 (3.4) [b]	11.0 (3.4)	11.0 (3.2)
**Trail Making Test A**	39.2 (15.4)	43.6 (19.7)	47.4 (19.0)	51.0 (20.3)
**Trail Making Test B**	93.3 (42.1)	107.2 (46.6) [b]	149.4 (71.7)	167.3 (78.5)
**Boston Naming Test**	27.8 (2.6)	27.7 (1.8)	25.5 (4.1)	25.5 (4.2)

a. FRS positivity (FRS+) was defined as the presence of ≥ 2 FRS at baseline. Those with one or no FRS were defined as FRS-. Characteristics were compared within diagnostic groups (i.e., Intact Cognition and Mildly Impaired) with Student's t test or chi-square statistics.

b. Within-group comparison significant at $p<0.05$

© 2026 Bojarski LG et al. *JAMA Network Open*.
