# Supplementary material for: Frontal Release Signs and Future Decline in Research Participants With Intact Cognition
Source: JAMA Netw Open. 2026 Jun 5;9(6):e2617060. doi: 10.1001/jamanetworkopen.2026.17060 (PMC13241946; doi:10.1001/jamanetworkopen.2026.17060)
Supplement: Supplement 2. — Data Sharing Statement [file jamanetwopen-e2617060-s002.pdf]

Data Sharing Statement

Bojarski. Frontal Release Signs and Future Decline in Research Participants With Intact Cognition. *JAMA Netw Open*. Published online June 5, 2026. doi:10.1001/jamanetworkopen.2026.17060

Data
Data available: Yes
Data types: Deidentified participant data
How to access data: Erin Abner erin.abner@uky.edu **When available:** With publication

Supporting Documents
Document types: None

Additional Information
Who can access the data: Requested data that has been approved
Types of analyses: for repetition of study
Mechanisms of data availability: signed data agreement will be required to access data
